# Mercury Content and Screening Exposure Assessment in Sports Food Supplements

**DOI:** 10.3390/toxics14080680

**Published:** 2026-08-01

**Authors:** Anna Puścion-Jakubik, Dominika Jabłonka, Daria Wojciuk, Renata Markiewicz-Żukowska, Katarzyna Socha

**Affiliations:** Department of Bromatology, Faculty of Pharmacy with the Division of Laboratory Medicine, Medical University of Białystok, Mickiewicza 2D Street, 15-222 Białystok, Poland; 39641@student.umb.edu.pl (D.J.); 46913@student.umb.edu.pl (D.W.); renata.markiewicz-zukowska@umb.edu.pl (R.M.-Ż.); katarzyna.socha@umb.edu.pl (K.S.)

**Keywords:** mercury, food supplements, food safety, sports supplements, exposure assessment

## Abstract

Sport-related food supplements are widely used by physically active individuals and athletes, but their chemical safety may vary with formulation, raw-material origin, and manufacturing practices. This study determined the mercury (Hg) content in 50 selected sports-related food supplements available on the Polish market and performed a screening exposure assessment based on the maximum manufacturer-recommended daily portion. Total Hg content was measured by atomic absorption spectrometry using an Advanced Mercury Analyzer AMA-254. The median Hg content was 9.008 µg/kg (interquartile range: 5.287–21.938 µg/kg; range: 0.395–51.694 µg/kg), and no statistically significant differences were found among supplement categories. All concentrations were below the European Union maximum level of 0.1 mg/kg for food supplements. For a 100 kg adult, all product-specific Target Hazard Quotient (THQ) values were below 1 in the hypothetical 100% inorganic Hg scenario. In the conservative 100% methylmercury scenario, three gel products with maximum recommended intakes of 300 g/day yielded THQ values of 1.040–1.551, although their contributions to the tolerable weekly intake remained below 100% (55.98–83.51%). Because only total Hg was determined, these calculations represent bounding screening scenarios rather than species-specific risk estimates. The findings demonstrate that regulatory compliance based on Hg concentration should be complemented by serving-size-based exposure assessment and support continued monitoring and direct Hg speciation in high-consumption sports supplements.

## 1. Introduction

Food supplementation has become an integral part of sports nutrition, with an increasing number of athletes relying on various products to support performance, endurance, and recovery. A meta-analysis of 128 studies found that approximately 60% of athletes reported using dietary supplements [1]. Confirming the high prevalence of supplement use in Poland, a nationwide cross-sectional study of 659 Polish athletes found that 91.1% reported using supplements, regardless of performance level or gender [2].

Under Polish law, a dietary supplement is defined as a foodstuff intended to supplement the normal diet and constituting a concentrated source of vitamins, minerals, or other substances with a nutritional or physiological effect, marketed in dose form [3]. Within the European Union, food supplements are regulated as foods rather than medicinal products [4]. Detailed requirements concerning their composition and labeling in Poland are specified in the Regulation of the Minister of Health on the composition and labelling of food supplements [5]. These regulations define permitted nutritional forms, product formulation rules, safety criteria, and mandatory labeling statements. Furthermore, European Commission Regulation (EU) 2023/915 establishes maximum levels for selected contaminants in food; for Hg in food supplements, the maximum level is 0.1 mg/kg [6].

Hg is one of the most toxic environmental pollutants. Numerous publications indicate that it has neurotoxic, nephrotoxic, hepatotoxic, cardiotoxic, reproductive, and immunotoxic effects [7,8,9,10,11,12,13,14].

Hg occurs in the environment in three basic forms: elemental (Hg^0^), inorganic (Hg^2+^), and organic, mainly as methylmercury (MeHg) [7,8]. These forms vary in bioavailability and toxicity, with MeHg posing the greatest concern following oral exposure because of its ability to bioaccumulate and biomagnify in the food chain [9]. Elemental mercury vapor can be absorbed through the respiratory tract, whereas inorganic forms, such as mercuric chloride, primarily affect the kidneys and liver [7,13]. MeHg is formed through the methylation of inorganic Hg by microorganisms in the aquatic environments and, after absorption, binds to sulfhydryl groups of proteins, inducing oxidative stress, mitochondrial dysfunction, and neuronal damage [12,15]. Its lipophilic nature allows it to cross the placental barrier and blood–brain barriers, posing a particular risk to fetal development [9].

Human exposure to Hg arises from both natural and anthropogenic sources. Hg is naturally released through volcanic activity, rock weathering, and geothermal processes, whereas industrial activities such as coal combustion, metal smelting, and gold mining are major contributors to global emissions [7,16,17]. In aquatic environments, microbial methylation converts inorganic Hg to methylmercury (MeHg), which bioaccumulates in fish and seafood, making diet the primary exposure route for most of the population [8,9]. Because Hg occurs naturally in the Earth’s crust and atmosphere, complete avoidance of exposure is impossible, and even products perceived as safe, such as dietary supplements, can contribute to increased exposure to this toxic component [13].

Many studies have investigated toxic elements, including Hg, in food supplements available on the market [18,19,20,21,22,23,24,25]. These studies generally reported Hg concentrations below the maximum level of 0.1 mg/kg, although isolated exceedances have been identified, for example, in preparations containing bamboo shoots or the alga *Chlorella pyrenoidosa* [23]. Most published investigations have focused on selected supplement categories, such as herbal preparations, fish oils, or protein powders, rather than on the diverse range of products marketed to physically active consumers.

Published data on Hg exposure from the broad range of sport-related supplements remain limited. Therefore, this study aimed to determine total Hg in selected sport-related food supplements available on the Polish market, compare Hg concentrations among product categories, assess compliance with the applicable maximum level, and estimate product-specific exposure using maximum manufacturer-recommended daily portions. Because Hg speciation was not performed, exposure was assessed using two hypothetical bounding scenarios: 100% of total Hg as inorganic Hg (iHg) and 100% as methylmercury (MeHg).

## 2. Materials and Methods

### 2.1. Materials

The study included 50 selected food supplements targeted at athletes and available on the Polish market. Products were purchased from physical stores and online retailers in 2025 and 2026 using purposive market sampling.

The sample comprised the following categories: non-stimulating ergogenic supplements (*n* = 5), pre-workout supplements with caffeine (*n* = 15), energy gels (*n* = 7), electrolyte supplements (*n* = 3), protein supplements (*n* = 10), collagen-containing supplements (*n* = 3), fat-burning supplements (*n* = 3), and amino acid supplements (*n* = 4).

The 50 products represented 14 declared brands/manufacturers. Because sampling was purposive and several products originated from the same brand/manufacturer, the dataset should be regarded as a market survey and not a probability-based sample representative of the entire Polish or European Union market.

### 2.2. Sample Preparation

Liquid dietary supplement samples were mixed using a Vortex Mixer Benchmixer (Benchmark, Sayreville, NY, USA). Solid dietary supplement samples were homogenized using a vortex mill (Testchem, Radlin, Poland). Aliquots of 50 µL of liquid samples or approximately 0.02 g of solid samples were placed in nickel cuvettes for Hg determination.

### 2.3. Determination of Hg

Hg content was determined by atomic absorption spectrometry (AAS) using an Advanced Mercury Analyzer AMA-254 (LECO Corp./Altec Ltd., Prague, Czech Republic). This method is based on thermal decomposition of the sample, separation of Hg from inorganic and organic compounds, and its conversion to its atomic form.

The determination procedure consisted of three main steps: drying the sample and subjecting it to thermal decomposition in a stream of oxygen, transporting the Hg vapor through a catalytic column for capture on a gold amalgamator, and then releasing it from the amalgamator and quantifying it by AAS at a wavelength of 253.7 nm. The instrumental limit of detection (LOD) was 0.003 ng Hg.

Method quality control was performed using the certified reference material Mixed Polish Herbs (INCT-MPJ-2), obtained from the Institute of Nuclear Chemistry and Technology (Warsaw, Poland). The analytical procedure applied to the reference material was identical to that used for Hg determination in the tested samples. The recovery rate was 103%, and the precision, expressed as the relative standard deviation (RSD), was 2.2%.

### 2.4. Assessment of Consumption Safety Based on Standards and Exposure Indicators

The exposure assessment was based on the total Hg concentration in each product and the maximum daily portion recommended by its manufacturer. When the label specified a range of permitted daily intakes, the upper value was selected to provide a high-intake screening scenario. For liquid products, the recommended volume was converted to mass, assuming a density of 1.0 g/mL, because product-specific densities were not determined.

A body weight (BW) of 100 kg was used as a predefined adult-athlete scenario. This value was selected to reflect a high-body-mass adult within the intended consumer population; because the calculated indices are inversely proportional to BW, the estimates should not be considered conservative for consumers weighing less than 100 kg.

The Estimated Daily Intake (EDI) and Estimated Weekly Intake (EWI) of Hg were calculated using the following equations:

EDI = (C × DI)/BW,(1)

EWI = EDI × 7,(2)

where EDI is the estimated daily intake of Hg expressed as µg/kg body weight/day, EWI is the estimated weekly intake expressed as µg/kg body weight/week, C is the total Hg concentration in the product (µg/kg), DI is the maximum manufacturer-recommended daily intake of the product (kg/day), and BW is the body weight (kg). Product-specific values were calculated first, and arithmetic means were subsequently calculated for each supplement category and for the total group.

The percentage contribution to the tolerable weekly intake (%TWI) was calculated as:

%TWI = (EWI/TWI) × 100(3)

The calculated EWI values were compared with the tolerable weekly intake values established by the European Food Safety Authority (EFSA): 4 µg/kg body weight/week for iHg and 1.3 µg/kg body weight/week for MeHg, both expressed as Hg [26].

Because only total Hg was measured, two hypothetical bounding scenarios were evaluated. The first assumed that 100% of total Hg was present as iHg, whereas the second assumed that 100% was present as MeHg. These scenarios were used for screening purposes and do not represent analytical Hg speciation.

The non-carcinogenic screening risk was evaluated using the Target Hazard Quotient (THQ):

THQ = EDI/RfD,(4)

where RfD is the oral reference dose expressed as µg/kg body weight/day. RfD values of 0.3 µg/kg body weight/day for iHg and 0.1 µg/kg body weight/day for MeHg were applied [27,28]. A THQ below 1 indicates that the exposure is unlikely to be associated with an appreciable non-carcinogenic health risk, whereas a THQ equal to or above 1 identifies a screening-level concern that requires further evaluation and does not by itself demonstrate an adverse health effect.

To illustrate the potential effect of concurrent supplement use, two additional sensitivity scenarios were calculated by multiplying the mean product-specific daily Hg intake by three and by twelve, corresponding to the median and upper range of simultaneous supplement use reported in athletes [29]. These calculations are illustrative cumulative-use scenarios and do not represent observed individual product combinations.

### 2.5. Statistical Analysis

Statistical analysis was performed using Statistica 13.3 software (TIBCO Software Inc., Palo Alto, CA, USA). Data distribution was assessed using the Shapiro–Wilk, Kolmogorov–Smirnov, and Lilliefors tests.

Descriptive statistics included the average (Av.) with standard deviation (SD), minimum (Min.), and maximum (Max.) values to enable comparison with previously published data. Due to the non-normal distribution of the data, median (Me), lower quartile (Q1) and upper quartile (Q3) values were also included.

Exposure and screening-risk indicators were calculated individually for each product and summarized using arithmetic means for each category and for the total group.

Differences in Hg concentration among the eight supplement categories were evaluated using the Kruskal–Wallis test. Because the test was not statistically significant, no post hoc pairwise comparisons were performed. Statistical significance was set at *p* < 0.05.

## 3. Results

### 3.1. Total Hg Concentration Across Supplement Categories

Hg concentrations were not normally distributed, so their interpretation was based primarily on median values and interquartile ranges. In the total study group, the median Hg content was 9.008 µg/kg, with an interquartile range of 5.287–21.938 µg/kg and a full range of 0.395–51.694 µg/kg (Table 1).

The highest median Hg content was observed in electrolyte supplements (Me = 28.772 µg/kg), followed by amino acid supplements (Me = 22.324 µg/kg), fat-burning supplements (Me = 18.890 µg/kg), non-stimulating ergogenic supplements (Me = 14.110 µg/kg), and collagen-containing supplements (Me = 10.608 µg/kg). Lower medians were observed in pre-workout supplements with caffeine (Me = 9.089 µg/kg), protein supplements (Me = 6.969 µg/kg), and energy gels (Me = 6.867 µg/kg).

The widest interquartile ranges were observed for energy gels (Q1–Q3: 2.397–41.136 µg/kg), amino acid supplements (11.205–33.132 µg/kg), and non-stimulating ergogenic supplements (14.033–28.541 µg/kg), indicating substantial within-category variability. In contrast, protein supplements showed a narrower interquartile range (5.270–7.786 µg/kg).

Although energy gels had one of the lowest category medians, this group contained the highest individual Hg concentration recorded in the study (51.694 µg/kg). Pre-workout supplements with caffeine also showed a wide range, from 0.395 to 42.617 µg/kg. Thus, low category medians did not exclude comparatively high concentrations in individual products.

The Kruskal–Wallis test showed non-statistically significant differences in Hg concentration among the eight supplements categories. Therefore, the category-specific medians should be interpreted descriptively and not as evidence of category-related differences in Hg concentration.

Table 2 presents descriptive total Hg concentrations in food supplements stratified according to the presence or absence of selected ingredients and formulation characteristics. No inferential comparisons were performed for these exploratory subgroups; therefore, the values should not be interpreted as evidence of ingredient-related differences in Hg concentrations.

### 3.2. Exposure and Screening Risk Assessment

All measured Hg concentrations were below the maximum level of 0.1 mg/kg applicable to food supplements in the European Union [6].

Product-specific exposure was calculated using a maximum manufacturer-recommended daily portion and a body weight of 100 kg. Recommended daily portions, converted to mass where necessary, ranged from 10.8 to 300 g/day, and the overall mean was 88.14 g/day. The iHg- and MeHg-based calculations represent hypothetical bounding screening scenarios because Hg speciation was not performed.

Across all 50 products, the mean EDI was 0.016 µg/kg body weight/day and the mean EWI was 0.111 µg/kg body weight/week. The mean THQ values were 0.053 for the 100% iHg scenario and 0.158 for the 100% MeHg scenario, while the corresponding mean contributions to the TWI were 2.766% and 8.511%, respectively (Table 3).

At the product level, all THQ values were below 1 in the 100% iHg scenario; the maximum THQ was 0.517 and the maximum contribution to the TWI was 27.140%. In the 100% MeHg scenario, three gel products—two energy gels and one electrolyte gel—had THQ values above 1 (range: 1.040–1.551). All three had a maximum manufacturer-recommended daily portion of 300 g. Their contributions to the MeHg TWI ranged from 55.976% to 83.506%, and no product exceeded 100% of the TWI.

In the illustrative cumulative-use analysis, the three-supplement scenario yielded THQ values of 0.158 for iHg and 0.474 for MeHg, corresponding to 8.298% and 25.532% of the respective TWI values. In the twelve-supplement scenario, the corresponding THQ values were 0.632 and 1.897, while the %TWI values were 33.191% for iHg and 102.127% for MeHg. Thus, the upper cumulative-use scenario exceeded both the MeHg RfD-based THQ threshold and the MeHg TWI, although it should be interpreted only as a sensitivity analysis.

The highest category mean exposure estimates were observed for energy gels (mean EDI: 0.052 µg/kg body weight/day; mean MeHg-based THQ: 0.520) and electrolyte supplements (mean EDI: 0.037 µg/kg body weight/day; mean MeHg-based THQ: 0.366). These category means remained below 1, but they did not capture the higher product-specific THQ values observed for the three high-serving-size gel products.

The results indicate that serving size materially affected the exposure assessment: products complying with the concentration-based maximum level could still reach a screening-level THQ of 1 or higher under the conservative assumption that all measured Hg was MeHg.

A THQ equal to or above 1 should not be interpreted as proof of an adverse health effect. In the present study, it identifies products warranting more refined assessment, particularly direct Hg speciation and verification of actual consumption patterns.

## 4. Discussion

The present study showed that Hg was detectable in all analyzed categories of sports-related food supplements, although the measured concentrations were generally low. The highest individual Hg concentration was 51.694 µg/kg, and all products complied with the European Union maximum level of 0.1 mg/kg for Hg in food supplements [6].

Although category medians varied numerically, the Kruskal–Wallis test did not show statistically significant differences among supplement categories. The category ranking should therefore be interpreted descriptively. Variability among individual products may reflect differences in ingredient composition, raw-material origin, and processing, but these potential determinants were not directly investigated in the present study [18,23,24].

In this study, Hg concentrations in the analyzed food supplements for athletes remained below the maximum permissible level specified in the Commission Regulations (EU) 2023/915 [6]. Similar observations have been published by other authors. For example, an analysis of dietary supplements containing vegetable and fish oils available on the Polish market showed Hg concentrations ranging from 0.023 to 0.427 µg/kg, with none of the analyzed products exceeding the applicable minimum level of 0.1 mg/kg [19]. Similarly, studies evaluating whey protein supplements have shown very low Hg concentrations, ranging from 0.548 to 9.41 ng/g, remaining well below the maximum limits established for food products [30]. Risk assessments conducted for protein powder supplements suggest that the ingestion of heavy metals, including Hg, is unlikely to cause adverse health effects when calculated hazard ratios generally remain below levels of concern [24]. Differences among studies should be interpreted in light of product composition, geographic origin, analytical methodology, and serving size.

Although the Hg concentrations detected in the analyzed supplements were low, the presence of trace amounts indicates that food supplements may contribute to overall exposure. The Hg detected in food supplements may originate from contaminated botanical or mineral raw materials, environmental conditions at the source, and variability introduced during processing and manufacture [18,23,24]. However, the present study did not analyze individual ingredients or production stages and therefore cannot identify the source of Hg in any specific product. Thus, continued monitoring of toxic-element contamination in food supplements remains warranted even when concentrations comply with regulatory limits.

Another important aspect when evaluating potential exposure to contaminants is the simultaneous use of multiple dietary supplements. Survey-based studies indicate that athletes frequently consume several products concurrently. For instance, an international study involving young athletes reported that 82.2% of participants used sports supplements, most commonly protein products [31]. Similarly, research among high-performance athletes showed that 64% of respondents used dietary supplements, with a median intake of three products and a range extending from one to twelve supplements used simultaneously [29]. Data derived from doping control forms further indicate that approximately half of athletes report supplement use, with an average of about two products taken concurrently [32]. Such supplementation patterns may increase cumulative exposure to trace contaminants, even when the concentration of a given element in a single product remains low. In the illustrative scenario based on three products at the mean product-specific exposure, both iHg- and MeHg-based indices remained below the screening thresholds. In contrast, the twelve-product scenario yielded a MeHg-based THQ of 1.897 and 102.127% of the MeHg TWI. These calculations illustrate the potential importance of cumulative exposure but should not be interpreted as estimates for a specific consumer because the model did not incorporate observed product combinations, intake frequency, or background dietary Hg exposure.

The exposure assessment indicated that, at the group level, mean Hg exposure and screening-risk indicators remained below the adopted screening thresholds when maximum manufacturer-recommended daily portions and a body weight of 100 kg were used. However, three gel products had MeHg-based THQ values of 1.040–1.551. All three products had maximum recommended daily portions of 300 g, demonstrating that the product-specific screening results depended on both Hg concentration and the amount consumed. Their MeHg-based %TWI values remained below 100% (55.98–83.51%). The simultaneous occurrence of THQ ≥ 1 and %TWI < 100% reflects the use of different health-based reference values—the U.S. EPA daily RfD and the EFSA weekly TWI—and the metrics should be interpreted independently [26,27,28]. Neither metric represents a probability of harm. Actual exposure also depends on the frequency and duration of use and the concurrent use of multiple products.

Only total Hg was determined in this study. Inorganic Hg and MeHg differ in toxicokinetics and toxicity [7,8,9,12,13], and the chemical form of Hg cannot be inferred from the supplement matrix alone. Therefore, the calculations based on reference values for inorganic Hg and MeHg represent two hypothetical bounding scenarios rather than species-specific exposure estimates. Direct Hg speciation would be required to determine the contribution of individual Hg species and to refine the toxicological assessment.

Future studies should therefore consider analyzing multiple toxic elements in a broader range of sport dietary supplements. Expanding the number and diversity of tested products would allow for a more comprehensive assessment of potential contamination. Furthermore, the determination of specific Hg species and continued monitoring of heavy metal levels in food supplements would help to better characterize the potential health risks associated with their long-term consumption.

Despite the valuable insights provided by this study, several limitations should be acknowledged. The results should also be interpreted in the context of the chemical form of Hg. Only total Hg was determined in this study. iHg and MeHg differ in toxicokinetics and toxicity [7,9,12,13], and the chemical form of Hg cannot be inferred from the supplement matrix alone. Thus, iHg- and MeHg-based calculations are hypothetical lower and upper toxicity screening bounds rather than species-specific exposure estimates. Direct speciation, for example, using an appropriate chromatographic separation coupled with element-specific detection, is required to determine the actual contribution of individual Hg species.

Several additional limitations should also be considered. The products were selected purposively and represented 14 brands/manufacturers, with some manufacturers contributing multiple products. Therefore, brand-level clustering may have affected the observed variability, and the sample should not be considered representative of the entire Polish or European Union market. Batch-to-batch variability was not assessed. Maximum label-recommended portions may differ from actual consumer intake, and the assumed density of 1.0 g/mL for liquid products introduces additional uncertainty. Moreover, using a BW of 100 kg yields lower body-weight-normalized exposure estimates than would be obtained for a lighter consumer. The illustrative cumulative-use scenarios did not reflect observed individual product combinations, intake frequency, or background Hg exposure from food and other environmental sources.

Future studies should include a broader and more systematically sampled range of sports supplements, multiple production batches, measured product densities, and detailed consumption data. Direct Hg speciation is particularly important for the three products with MeHg-based THQ values above 1. Parallel determination of other toxic elements would also provide a more comprehensive assessment of the chemical safety of sports-related supplements.

## 5. Conclusions

All analyzed sport-related food supplements complied with the European Union maximum level for total Hg, and the category-level mean exposure indicators were below the adopted screening thresholds. Nevertheless, under the conservative assumption that 100% of total Hg was MeHg, three gel products with maximum recommended intakes of 300 g/day had THQ values of 1.040–1.551. No individual product exceeded 100% of the TWI. An illustrative twelve-supplement cumulative-use scenario also exceeded the MeHg-based THQ threshold and reached 102.127% of the MeHg TWI. These findings do not demonstrate an actual MeHg-related risk because Hg speciation and individual consumption patterns were not determined, but they show that concentration-based compliance alone may not fully characterize exposure when daily serving sizes are large or several products are used concurrently. Continued monitoring, product-specific exposure assessment, cumulative-use modelling, and direct Hg speciation are warranted.

## Figures and Tables

**Table 1:** Total Hg concentrations in the analyzed categories of sport-related food supplements.

Type	Av. ± SD	Min–Max	Me	Q1–Q3
non-stimulating ergogenic supplements	19.533 ± 10.177	8.837–32.145	14.110	14.033–28.541
pre-workout supplements with caffeine	11.913 ± 11.591	0.395–42.617	9.089	1.379–16.995
energy gels	20.498 ± 20.829	1.849–51.694	6.867	2.397–41.136
electrolyte supplements	22.829 ± 15.664	5.062–34.652	28.772	5.062–34.652
protein supplements	6.482 ± 1.737	3.134–8.752	6.969	5.270–7.786
collagen-containing supplements	10.494 ± 3.905	6.534–14.342	10.608	6.534–14.342
fat-burning supplements	18.187 ± 4.172	13.709–21.963	18.890	13.709–21.963
amino acid supplements	22.168 ± 17.591	0.472–43.554	22.324	11.205–33.132
Total	14.558 ± 12.829	0.395–51.694	9.008	5.287–21.938

Av—average, Max—maximum, Me—median, Min—minimum, Q1—lower quartile, Q3—upper quartile, SD—standard deviations.

**Table 2:** Descriptive total Hg concentrations according to the presence or absence of selected ingredients and formulation characteristics.

Ingredients	Presence	*n*	Av. ± S D	Min–Max	Me	Q1–Q3
Plant-Based Supplements	Yes	17	12.746 ± 11.269	0.395–42.617	11.399	1.427–18.890
	No	33	15.491 ± 13.633	0.472–51.694	8.837	6.533–22.709
Nootropics	Yes	16	12.533 ± 10.970	0.395–42.617	10.244	1.427–18.890
	No	34	15.696 ± 13.799	0.472–51.694	8.794	5.910–25.625
Guarana	Yes	9	10.163 ± 10.005	0.395–26.171	9.089	1.096–16.995
	No	41	15.522 ± 13.277	0.472–51.694	8.926	6.608–21.938
Capsaicin	Yes	2	9.643 ± 13.077	0.395–18.890	9.643	0.395–18.890
	No	48	14.762 ± 12.918	0.472–51.694	9.008	5.911–21.951
Caffeine	Yes	15	12.608 ± 7.876	0.395–26.171	14.033	6.867–18.890
	No	35	15.393 ± 14.465	0.472–51.694	8.752	5.270–28.541
Taurine	Yes	13	17.238 ± 14.949	0.395–51.694	14.033	8.706–20.115
	No	37	13.616 ± 12.083	0.472–43.554	8.752	5.270–16.667
Tyrosine	Yes	4	9.281 ± 9.881	0.395–20.115	8.308	0.911–17.651
	No	46	15.016 ± 13.038	0.472–51.694	9.008	6.534–21.963
Citrulline	Yes	9	19.201 ± 12.210	8.837–42.617	14.110	9.089–28.541
	No	41	13.538 ± 12.879	0.395–51.694	8.180	5.127–20.115
Vitamin C	Yes	10	11.120 ± 9.212	1.096–28.772	11.004	1.427–15.568
	No	40	15.417 ± 13.543	0.395–51.694	8.882	6.571–22.336
Vitamin B	Yes	16	16.338 ± 11.799	0.670–34.652	14.852	5.882–27.356
	No	34	13.720 ± 13.372	0.395–51.694	8.729	11.708 –16.995
Adaptogens	Yes	4	16.266 ± 13.201	1.427–28.541	17.549	5.177–27.356
	No	46	14.409 ± 12.934	0.395–51.694	8.963	5.287–20.115
Natural Colors	Yes	18	11.940 ± 10.817	1.427–43.554	7.565	5.270–15.188
	No	32	16.029 ± 13.774	0.395–51.694	12.158	6.617–24.440
Synthetic Colors	Yes	21	16.016 ± 13.980	0.395–43.554	8.180	5.287–21.938
	No	29	13.501 ± 12.069	0.472–51.694	10.608	6.534–15.188
Natural Sweeteners	Yes	8	17.557 ± 12.938	0.395–34.652	14.197	7.814–30.693
	No	42	13.986 ± 12.885	0.472–51.694	8.795	5.270–20.115
Synthetic Sweeteners	Yes	40	15.700 ± 12.695	0.395–51.694	12.158	6.969–21.950
	No	10	9.986 ± 12.984	0.670–34.652	4.549	1.379–11.399

Av—average, Max—maximum, Me—median, Min—minimum, Q1—lower quartile, Q3—upper quartile, SD—standard deviations.

**Table 3:** Mean exposure and screening-risk indicators for sport-related food supplements, calculated using the maximum manufacturer-recommended daily portion and a body weight of 100 kg.

Type	Average Serving Size [g/Day]	EDI	EWI	THQ (iHg)	%TWI (iHg)	THQ (MeHg)	%TWI (MeHg)
non-stimulating ergogenic supplements	80.00	0.015	0.104	0.049	2.597	0.148	7.992
pre-workout supplements with caffeine	73.27	0.007	0.052	0.025	1.306	0.075	4.018
energy gels	165.71	0.052	0.364	0.173	9.097	0.520	27.991
electrolyte supplements	111.67	0.037	0.256	0.122	6.409	0.366	19.721
protein supplements	105.00	0.007	0.048	0.023	1.191	0.068	3.665
collagen-containing supplements	11.03	0.001	0.008	0.004	0.201	0.011	0.691
fat-burning supplements	60.00	0.011	0.075	0.036	1.885	0.108	5.800
amino acid supplements	37.50	0.007	0.046	0.022	1.162	0.066	3.576
Total	81.14	0.016	0.111	0.053	2.766	0.158	8.511

EDI—Estimated Daily Intake, EWI—Estimated Weekly Intake, THQ—Target Hazard Quotient, TWI—Tolerable Weekly Intake.

## Data Availability

The data supporting the findings of the study are included in the article. Further inquiries may be directed to the corresponding author.

## Abbreviations

The following abbreviations are used in this manuscript:

Hg	Mercury
iHg	Inorganic mercury
MeHg	Methylmercury
EDI	Estimated Daily Intake
EWI	Estimated Weekly Intake
RfD	Reference dose
THQ	Target Hazard Quotient
TWI	Tolerable Weekly Intake
